# Student Perceptions on Educational Handovers from Medical School to Residency

**DOI:** 10.1007/s40670-026-02856-8

**Published:** 2026-08-15

**Authors:** Lauren S. Starnes, Amy E. Fleming, Kendra Parekh

**Affiliations:** 1 Department of Pediatrics, Division of Pediatric Hospital Medicine, Vanderbilt University Medical Center, 2141 Blakemore Avenue, Nashville, TN 37212 USA; 2 Vanderbilt University School of Medicine, Nashville, TN USA; 3 Department of Emergency Medicine, Vanderbilt University Medical Center, Nashville, TN USA

**Keywords:** Transition to residency, Educational handover, UME to GME transition, Onboarding

## Abstract

To improve the transition to residency, the Coalition for Physician Accountability recently recommended a post-Match, learner-driven educational handover. Few studies have analyzed students’ perspectives on the content of handovers, especially the inclusion of personal values or self-care goals. We surveyed graduating medical students at our institution on their perspectives about the content in an educational handover to residency programs. A subset agreed with sending goals, personal values, and learning style to program directors. Students expressed concerns with sending feedback or grades and the potential to perpetuate prior perceptions.

## Background

Trainees find the transition from undergraduate medical education (UME) to graduate medical education (GME) to be an unexpected change in roles and responsibilities [1]. Likewise, residency program directors report gaps between interns’ skills and what is expected of them [2–4]. In 2021, the Coalition for Physician Accountability’s UME-GME Review Committee (UGRC) developed 34 recommendations to improve the transition to residency [5] including exchanging updated information on student performance, or educational handovers, between medical schools and residency programs after Match. However, a standardized format or structure has yet to be developed.

Little is known about students’ perceptions of educational handovers [6] and their content related to the transition to residency despite being key stakeholders. There has been little exploration into whether learners would support inclusion of holistic components beyond academic metrics in a handover. Most research has focused on the process of communicating performance data between medical schools and residency programs or on program directors’ feedback on handovers [7–10]. However, understanding learner perspectives is critical, as student acceptance and trust of a handover from medical school to residency is likely to influence the success of implementing this UGRC recommendation.

As medical education has shifted towards competency-based assessment, institutions have increasingly used learning portfolios and academic coaches to provide space for aggregating and analyzing performance measures [11]. These supports for continued growth emphasize learner agency and self-regulated learning [12]. Specifically, our institution approaches our portfolio coaching program and learner development using the Master Adaptive Learner (MAL) framework. This framework explains learning as a self-regulated process that occurs through iterative cycles of identifying a learning need and establishing a goal (planning), interacting with resources (learning), practicing learned information in the context of feedback (assessing), and reflecting on ways to change practice moving forward (adjusting) [12, 13]. Our students repeatedly engage in goal-setting cycles during their training through reflection of learning portfolio content and with the assistance of their academic coach. These iterative cycles support continual adaptation in a complex working and learning environment, instilling self-regulation skills vital for transition processes [12]. 

During the transition to residency, sharing information about a learner’s goals and personal information may help program directors better support iterative learning cycles of new interns. We sought to apply the MAL framework and support our students’ agency in the transition to residency by seeking their perspectives on the content of handovers as related to goal-setting and information captured in their learning portfolios.

## Activity

In March 2024, we distributed an anonymous online survey to our institution’s graduating medical students. On a five-point scale (strongly disagree to strongly agree), students rated their agreement with including personal values, self-care goals, learning style, and clinical/academic goals in a student-generated handover. Students also rated their agreement with including self-care goals, clinical/academic goals, written rotation feedback, and grades in a medical school-generated handover. These categories of data reflect information available in our students’ learning portfolios and are commonly present in coaching portfolios used in medical schools across the country [11]. In open-ended responses, students provided any additional content they would like included in an educational handover. We used descriptive statistics to describe ratings and inductively coded open-ended responses for themes, discussing until consensus was reached. This study was approved by the Vanderbilt University Institutional Review Board (IRB #240038).

## Results & Discussion

### Quantitative Findings

80% (74/93) of students responded. For student-generated handovers, a simple majority agreed/strongly agreed with including clinical/academic goals (*n* = 46, 62%), learning style (*n* = 44, 59%), and personal values (*n* = 40, 54%). Regarding school-generated handovers, over half of students responded favorably about including clinical/academic goals (*n* = 43, 58%). Only 41% (*n* = 30) agreed/strongly agreed with sending written feedback and 17% (*n* = 13) with sending grades. Student agreement was mixed on including self-care goals in either type of handover (student-generated: *n* = 37, 50%; school-generated: *n* = 35, 47%).

### Qualitative Findings

In open-ended responses, students wrote they would like to include their areas of confidence, areas of improvement, and personal information (e.g., family information or religious commitments) as supporting information. Students expressed concerns that programs already received their academic record in the residency application and did not see a benefit in re-sending. One student wrote, “If they were good enough to rank me to Match, they do not need to be sent again.” Seven students (9.5%) provided general concerns about handovers. The most prominent theme was the potential to perpetuate perceptions from medical school to residency. One student wrote, “I strongly disagree with a learner handoff as it may facilitate preformed biases among residency leadership for the incoming resident, given the tough experiences and growth often experienced by students during medical school.” Another wrote, “I would much rather arrive to a new program without having medical school evaluations coloring the program’s expectations.” Others questioned the value of the handover, commenting that residency programs already learn about students through applications and interviews. Finally, students commented on concerns about the burden of developing a leaner-driven handover.

### Discussion

We identified four key perspectives from our students on educational handovers for the transition to residency. First, a simple majority of our students positively viewed including learner-generated clinical/academic goals, learning style, and personal values in an educational handover from medical school to residency programs after Match. This adds to prior work investigating student perspectives [6]. These findings support the inclusion of an individualized learning plan in an educational handover to facilitate the transition to residency [6, 10, 14]. This may enable program directors to intervene early in areas of growth and better support trainees as they transition.

Second, our students expressed mixed perceptions on inclusion of self-care goals. Others have found that students feel information about wellbeing is important in learner handovers [15]. As the format of educational handovers for the transition to residency becomes standardized, future qualitative work should be undertaken to better understand students’ views on holistic handovers to residency and whether information beyond performance in medical school should be included.

Third, a minority of students wanted grades and written feedback in a handover, and several expressed this was redundant from their residency application. This response may be implying that our students view Matching as an endpoint to medical school, despite applications including a fraction of the work done in the final year. A possible explanation of this is “preresidency syndrome,” or the excessive focus on obtaining a residency spot [16]. Once that spot is obtained, students may become less concerned with grades and feedback, making post-Match students less invested in sharing assessments with program directors.

Fourth, several students expressed concerns about handovers, especially their potential to perpetuate opinions of students into residency. Evidence suggests that handovers in a variety of settings have the potential to bias evaluators towards viewing learners as above or below average [17, 18]. In the context of transitioning to residency, this may be detrimental to both the learner and patients [19]. The impact of this must be considered as handovers are standardized.

This preliminary student survey has several limitations. We surveyed a single class of students at one institution, affecting generalizability. We did not ask students to state their intended specialty, and it may be that students pursuing different specialties have unique perceptions on the content to include in an educational handover. We also did not define categories of data (i.e., “learning style”) which may have impacted interpretation.

Our study provides foundational work for future efforts. We did not explicitly ask students whether they wanted to have an educational handover implemented, given that this was a UGRC recommendation. While many used open-ended responses to indicate that they preferred not to have one, future work should explore this with a more robust, validated survey and in-depth qualitative data. Future efforts should be expanded to include students from more than one institution and cohort, as well as early interns to investigate their perceptions on whether a handover did or could influence their transition to residency. Additionally, studies should include program directors to gather their perceptions on handovers and if or how they would utilize this content to support students’ transition to residency.

In conclusion, we performed an exploratory study to understand our students’ perspectives on educational handovers for the transition to residency that should inform future work. We found that graduating medical students favorably viewed sharing personal values, learning style, and clinical/academic goals but preferred not to include grades or written feedback in an educational handover to program directors. There were significant concerns about the utility, administrative burden, and potential implications of handovers.

## Data Availability

The datasets generated during and/or analyzed during the current study are available from the corresponding author on reasonable request.
